# Experimental Periodontitis Increases Anxious Behavior and Worsens Cognitive Aspects and Systemic Oxidative Stress in Wistar Rats

**DOI:** 10.1002/cre2.70017

**Published:** 2024-11-04

**Authors:** Giselle B. de Castro, Ramona R. S. Pereira, Caíque O. Diniz e Magalhães, Karine B. Costa, Etel R. Vieira, Ricardo C. Cassilhas, Kinulpe H. Sampaio, Alan R. T. Machado, Jaqueline do Carmo L. Carvalho, Ramiro M. Murata, Luciano J. Pereira, Marco F. Dias‐Peixoto, Eric F. Andrade, Vanessa Pardi

**Affiliations:** ^1^ Health Sciences Program Universidade Federal dos Vales do Jequitinhonha e Mucuri (UFVJM) Diamantina Minas Gerais Brazil; ^2^ Biological and Health Sciences Department Universidade Federal dos Vales do Jequitinhonha e Mucuri (UFVJM) Diamantina Minas Gerais Brazil; ^3^ Department of Exact Sciences Universidade do Estado de Minas Gerais João Monlevade Minas Gerais Brazil; ^4^ Department of Foundational Sciences, School of Dental Medicine East Carolina University (ECU) Greenville North Carolina USA; ^5^ Department of Health Sciences Universidade Federal de Lavras (UFLA) Lavras Brazil

**Keywords:** anxiety, cognition, oxidative stress, periodontal disease

## Abstract

**Objectives:**

Periodontitis (PD) has the potential to induce systemic changes that affect both physical and behavioral aspects. These alterations may be associated with changes in both the inflammatory profile and the oxidative stress status of individuals with PD. Therefore, we aimed to evaluate the effects of PD on oxidative stress, as well as on behavioral parameters and cognitive impairment, in a preclinical model.

**Material and Methods:**

Twenty‐four male Wistar rats were randomly assigned to PD and sham groups. PD was induced by the ligature protocol for 14 days. Behavioral tests were initiated on the 9th day of the experiment to evaluate anxious behavior and cognition (learning and memory). After euthanasia, oxidative stress was evaluated in the gums, blood, hippocampus, and amygdala. Alveolar bone loss, bone microstructure, and elemental compositions of the mandibular bone were also assessed.

**Results:**

PD increased alveolar bone loss, reduced the calcium and phosphorus content in the mandibular bone, and increased anxiety‐like behavior and cognitive decline (*p* < 0.05). Furthermore, PD significantly affected the redox balance, as evidenced by increased total antioxidant capacity (TAC) in the gingiva and hippocampus (*p* < 0.05). It also led to increased lipid peroxidation in the gingiva and erythrocytes (*p* < 0.05), decreased antioxidant defenses in erythrocytes (superoxide dismutase) and the hippocampus (catalase), and increased antioxidant activity (catalase) in the amygdala (*p* < 0.05).

**Conclusion:**

PD resulted in cognitive alterations, including impairments in spatial learning and memory, as well as increased anxious behavior, likely due to redox imbalance in rats.

## 1 Introduction

Periodontal disease (PD), also known as periodontitis, is an inflammatory condition that primarily affects the supportive tissues of teeth, triggering complex innate and adaptive inflammatory responses (Papapanou et al. 2018; Meyle and Chapple 2015). The disease initiates with the formation of biofilm in the periodontium, which, if left untreated, incites the host's inflammatory response, leading to increased levels of pro‐inflammatory mediators and oxidative stress markers. This inflammatory cascade ultimately promotes the resorption of alveolar bone (Kinane, Stathopoulou, and Papapanou 2017; Sczepanik et al. 2020). Furthermore, the translocation of periodontal pathogens into the systemic circulation is observed, potentially resulting in low‐grade subclinical inflammation—a factor epidemiologically linked to various conditions, including diabetes *mellitus*, cardiovascular diseases, and Alzheimer's disease (Hajishengallis 2022; Cai et al. 2021; Hajishengallis and Chavakis 2021).

Chronic low‐grade inflammation is a common feature in diseases such as diabetes *mellitus*, cardiovascular disorders, and nonalcoholic fatty liver disease, which have all been associated with cognitive changes and anxiety (Zilliox et al. 2016; Felger 2018). In this context, the chronic inflammation seen in PD shares features that may increase the risk of anxiety (Andrade et al. 2017) and cognitive impairment (Zheng et al. 2021; F. Liu et al. 2018; Li et al. 2022). Elevated oxidative stress and increased pro‐inflammatory cytokines in the brain are linked to cognitive decline (Zilliox et al. 2016; Gaspar et al. 2016). Meanwhile, a systemic pro‐inflammatory *status* can impact relevant brain regions, leading to symptoms such as reduced motivation and increased anxiety (Felger 2018). A systematic review observed that individuals with PD showed elevated levels of anxiety (Aragão et al. 2021). However, the mechanisms behind this relationship remain unclear. Chronic inflammation in PD may lead to the entry of periodontal pathogens into systemic circulation, triggering a pro‐inflammatory and pro‐oxidative cascade that could impact specific regions of the central nervous system (CNS) involved in behavior (Li et al. 2022; Martínez et al. 2022).

PD and behavioral disorders, including anxiety and cognitive impairment, have a high global prevalence (Papapanou et al. 2018; Nazir et al. 2020; Atabay et al. 2017). Furthermore, anxiety ranks as the second most debilitating condition worldwide, affecting approximately 374 million individuals in 2020, with a notable 26% increase over the past 2 years (Freeman 2022). Although the existing literature suggests a potential relationship between systemic inflammation stemming from PD and the development of cognitive (Hu et al. 2021) and behavioral changes (Andrade et al. 2017), further investigation with controlled variables to mitigate potential biases is essential (Felger 2018; Costello et al. 2019; Milaneschi et al. 2021). Therefore, evaluation of this relationship in preclinical models becomes paramount, as it allows for the control of factors such as disease exposure duration, as well as variables related to dietary habits and physical activity that can influence outcomes related to mental health and cognition.

Against this backdrop, we aimed to investigate the influence of PD on behavioral parameters related to anxiety and cognition. Additionally, we sought to explore the effects of PD on both local and systemic oxidative stress parameters in an experimental model of PD induced by a ligation protocol.

## 2 Materials and Methods

### 2.1 Ethical Considerations

This study was reviewed and approved by the Animal Use Ethics Committee of the Federal Universiade dos Vales do Jequitinhonha e Mucuri (UFVJM) under protocol number 24/2021. All procedures were carried out in accordance with the ethical principles established by the National Council for the Control of Animal Experimentation (CONCEA) and the guidelines of the Animal Research Reporting In Vivo Experiment (ARRIVE). The number of animals per group was kept to a minimum for ethical reasons but was still sufficient to achieve statistical significance. The sample size was determined to provide 80% statistical power to detect a significant difference of 20% in alveolar bone loss (ABL) between groups, with a standard deviation of 15%, and a 95% confidence interval (*α* = 0.05). Additionally, the sample size used is based on studies where PD was induced in Wistar rats (Andrade et al. 2017; de O Silva et al. 2017; V. O. Silva, Lobato, et al. 2015). Thus, 24 healthy adult male Wistar rats (*Rattus norvegicus albinus*) were obtained from the Laboratory of the Universidade Federal de Viçosa (UFV).

### 2.2 Animals and Experimental Conditions

During the experimental period, the rats were housed in collective polypropylene boxes (*n* = 4 per box) measuring 41 × 34 × 17.5 cm, lined with wood shavings, and under ideal conditions for the species (22 ± 2°C, humidity of 45 ± 15% and 12/12 h inverted light/dark light cycle). Water and food were provided ad libitum throughout the experiment.

**Figure 1:**
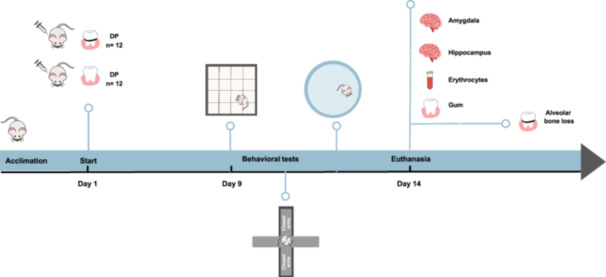
Experimental design.

Initially, the animals were subjected to 7 days of acclimatization to the experimentation room and researchers. Subsequently, the rodents were randomly distributed into two groups: PD group (PD; *n* = 12) and sham group (*n* = 12). PD was induced by the ligation protocol on the mandibular first molar on the first day of the experiment. On the ninth day after placing the ligature, the animals were subjected to behavioral tests (Morris Water Maze Test, Open Field Test, and Elevated Plus Maze) as described previously (Cassilhas, Lee, Venâncio, et al. 2012; Wellman et al. 1998; Pellow et al. 1985; Morris 1984). On the 14th day after PD induction, the animals were euthanized by guillotine decapitation. The steps of the experiment are shown in Figure 1.

### 2.3 PD Induction

PD was induced by the ligation protocol on the mandibular first molars as described by Messora et al. (2013). Subsequently, the animals were anesthetized by an intraperitoneal injection (13 mg/kg of 10% xylasin hydrochloride and 80 mg/kg of ketamine base), and then a cotton thread was placed around the first molar of each lower hemimandible. The ligature remained in place for 14 days until euthanasia. In the sham group, the anesthetic protocol was performed; however, the ligature was not placed.

### 2.4 Behavioral Tests

To assess anxious behavior, learning, and spatial memory, the elevated plus‐maze, open field, and Morris water maze tests were performed after the 9th day of PD induction. To enable adaptation to the test room, the animals were acclimated for 30 min before the start of the experiment and after each test, the apparatus was sanitized with 70% ethanol.

The open field was used to assess anxiety‐like behavior, as described by Wellman et al. (1998), and was carried out on the 9th day after PD induction. During the test, the distance covered by the animal was recorded and analyzed, in addition to the time spent in the central and peripheral regions of the apparatus. The elevated plus maze was performed on the 10th day after PD induction as described by Pellow et al. (1985). The time spent by each animal in the open arms, closed arms, and in the center was recorded.

On the 11th day after PD induction, the animals were tested in the Morris water maze to evaluate learning and memory parameters as described by Cassilhas, Lee, Fernandes, et al. (2012). Thus, during the training phase, which lasted 2 days, the rats were exposed to the Morris water maze four times (trials). Each rat randomly explored the water maze for 60 s. For the spatial acquisition test (spatial learning), the rats were subjected to three blocks of four trials with a 30‐min interval between the blocks. For the probe trial test, which was conducted 30 min after the spatial acquisition test, the platform was removed and the rats performed a single trial of 60 s. Escape latency and time spent on area were recorded. All behavior tests were recorded and analyzed using the Etho Vision Noldus XT V.16 video system (Leesburg, VA).

### 2.5 Euthanasia and Sample Collection

At the end of the experiment, the animals were euthanized by guillotine decapitation. Then, the hemimandibles were removed and bilaterally dissected. The hippocampus and amygdala were collected from the right hemispheres of the brain. Additionally, blood samples and the gums surrounding the mandibular first molar were collected. Gum, hippocampus, amygdala, and blood samples were kept on ice for fresh analysis as described previously (Costa et al. 2022). The hemimandibles were stored in 10% buffered formalin.

### 2.6 ABL Assessment

The right hemimandibles were dissected and submerged in hydrogen peroxide for 24 h. Then, the specimens were cleaned, dried, and stained with 1% methylene blue. The specimens were photographed using a stereoscopic magnifying glass and the digital images were used to evaluate ABL. Thus, linear measurements of the distance between the cemento–enamel junction (CEJ) and the alveolar bone crest (ABC) using the buccal surface were performed (de O Silva et al. 2017; Gusmão et al. 2021). ABL was determined by the average measurement of the three roots (Dai et al. 2016). Additionally, the area of resorption (mm^2^) corresponding to the exposed roots of the molars (without bone covering them) was assessed as described in a previous study (Martins et al. 2016). Measurements were taken using Image J software. All measurements were performed by a calibrated examiner (GBC) who was blinded to the experimental groups. Linear and area measurements of resorption between the CEJ and the alveolar bone crest were performed, with reference samples used to familiarize the examiner with anatomical landmarks. Repeated measurements on the same mandibles across multiple sessions ensured consistency, and after a 7‐day interval, the measured values were compared. The intraclass correlation coefficient (ICC) was calculated to assess intra‐examiner concordance, targeting a value of ≥ 0.75.

### 2.7 Morphological and Composition Analyses of Mandibular Bone by Scanning Electron Microscopy Coupled With Energy‐Dispersive Spectroscopy (SEM/EDS)

For the evaluation of bone microstructure and identification of possible alterations in the morphology of the mandibular bone, each mandible was placed on the surface of an aluminum holder using double‐sided carbon tape and analyzed by SEM (Vega 3 LMU, TESCAN, Brno‐Kohoutovice, Czech Republic). Images were obtained at magnifications of ×27, ×75, and ×500. Elemental compositions were determined using Energy‐Dispersive X‐ray Spectroscopy (EDS) with the X‐MaxN system (Oxford Instruments, Abingdon, United Kingdom). Spectra were obtained in an area, under an acceleration voltage of 20 kV, and a working distance of 13 mm. Data analysis was performed using AZtec 3.1 software (Oxford Instruments, Abingdon, United Kingdom). The percentages of calcium, phosphorus, carbon, and oxygen were determined, and the data were normalized by autoscaling; also, the Euclidean distance was used.

### 2.8 Redox Status Evaluation

Gum, hippocampus, amygdala, and blood samples were kept fresh for analysis as described previously (Costa et al. 2022). Tissue fragments were homogenized in a Potter–Elvehjem tissue grinder with ice‐cold PBS on an ice bath. Lipid peroxidation was assessed by quantifying thiobarbituric acid‐reactive substances (TBARS). The tissue homogenates were incubated with 0.8% thiobarbituric acid for 90 min at 90°C. Absorbance was measured at 532 nm for TBARS quantification, using a standard curve based on a malondialdehyde (MDA) solution (1,1,3,3‐tetramethoxypropane). The total antioxidant capacity (TAC) was assessed using the ferric‐reducing antioxidant power (FRAP) method (Benzie and Strain 1996). This method monitors the reduction of the Fe^3+^–ferric tripyridyltriazine (TPTZ) complex into Fe^2+^–TPTZ at an acidic pH, with absorbance measured at 593 nm. SOD activity was determined by monitoring the inhibition of pyrogallol autoxidation at 420 nm over 4 min at 37°C. For the quantification of carbonyl derivatives, proteins in homogenates were precipitated using 10% trichloroacetic acid and incubated with 2,4‐dinitrophenylhydrazine (DNPH, 10 mM) in 2 mM HCl at room temperature for 30 min, protected from light. The protein precipitate was washed twice with an ethanol/ethyl acetate (1:1) mixture and then dissolved in 6% sodium dodecyl sulfate. Samples were centrifuged at 10,000 g for 10 min at 4°C, and the supernatant was analyzed at 370 nm using the DNPH molar extinction coefficient of 22,000 M⁻¹ cm⁻¹. Catalase (CAT) activity was determined according to the method described previously (Nelson and Kiesow 1972). For this, the samples were added to a 0.03 M hydrogen peroxide solution (Sigma, USA) in quartz cuvettes and monitored for 60 s at 25°C, spectrophotometrically, at 240 nm using a dual‐beam UV‐visible spectrophotometer (CE‐Libra S22, Faotuo). The absorbance of hydrogen peroxide was recorded at 15, 30, 45, and 60 s during its decomposition reaction. Measurements were performed in triplicate, and CAT activity was expressed as millimoles of H_2_O_2_ decomposed per minute per milligram of protein (ΔE/min/mg prot.).

Protein content in the samples was quantified using the Bradford method (Bradford 1976). A standard curve was prepared using known concentrations of bovine serum albumin (BSA, 1 mg/mL). After mixing the samples with Coomassie Blue, the resulting color change, indicating protein binding, was measured. Absorbance readings were taken in triplicate at 590 nm using a spectrophotometer, and protein concentrations were calculated and expressed in mg/mL.

### 2.9 Statistical Analyses

The Shapiro–Wilk test was used to assess the normality of the data. Results are presented as mean ± standard deviation and were analyzed using Student's *t*‐test, one‐way ANOVA, or two‐way ANOVA with Tukey post hoc tests, as needed. Statistical analysis was conducted using GraphPad Prism version 8.0. Statistically significant differences were considered when *p* < 0.05.

## 3 Results

**Figure 2:**
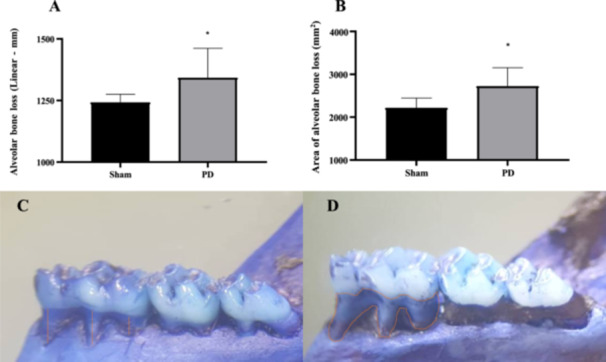
(A) Alveolar bone loss (ABL) was evaluated through the average linear distance between the cemento–enamel junction (CEJ) and the alveolar bone crest (AOC) of the three roots of the mandibular first molar, expressed in millimeters (mm). (B) Area of alveolar bone loss was assessed by measuring the area located between the CEJ and the AOC of the mandibular first molar, expressed in square millimeters (mm^2^). (C) Representation of the linear measurement region of ABL. (D) Representation of the area measurement region of ABL. *Significant difference assessed using an unpaired student's *t*‐test (*p* < 0.05).

ABL was greater in animals with PD both in the linear assessment and in the evaluation of the area of bone resorption (*p* < 0.01—Figure 2A,B).

**Figure 3:**
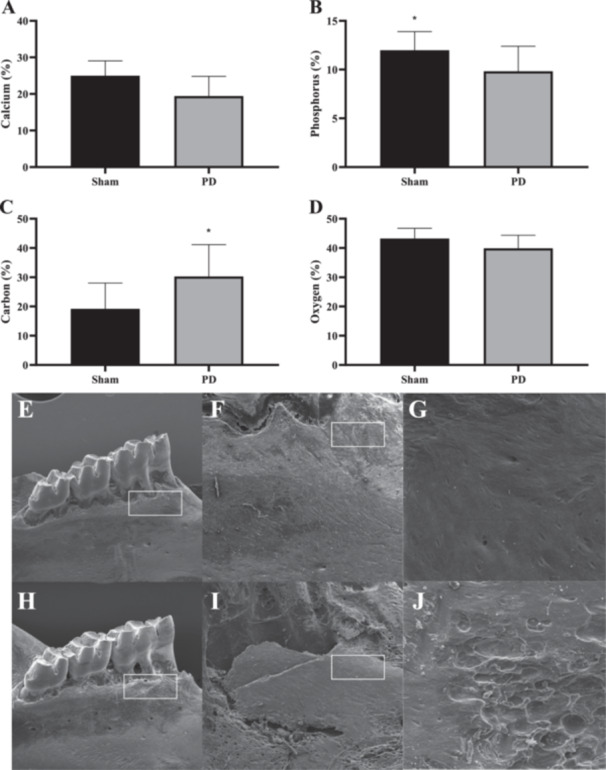
Elemental composition of the alveolar bone surface below the first mandibular molar in rats with ligature‐induced periodontal disease (PD). Values expressed in percentage ([A] calcium; [B] phosphorus; [C] carbon; [D] oxygen). Representation of the topography of the alveolar bone under the first molar at magnifications of ×27, ×75 and ×500. (E–G) Sham group. (H–J) PD group. The rectangles in images E, F, H, and I highlight the regions of interest that are shown in greater detail in the subsequent higher magnification images. *Significant difference by an unpaired student's *t*‐test (*p* < 0.05).

In terms of the elemental composition of the alveolar bone, we noted a reduction in the percentage of calcium and phosphorus in animals with PD compared with the sham group (*p* < 0.05, Figure 3A,B). The percentage of carbon on the surface of the alveolar bone was higher in the animals of the PD group (*p* < 0.05, Figure 3C). The percentage of oxygen on the surface of the alveolar bone did not differ between groups (*p* > 0.05, Figure 3D). Additionally, the analysis of the topography of the alveolar bone under the first mandibular molar revealed greater roughness and porosity of the bone surface in the animals of the PD group (Figure 3E–H).

Higher concentrations of TBARS and greater TAC were observed in the gum of animals with PD (*p* < 0.05, Table 1). No significant differences were observed between the groups for the values of carbonyl derivatives, SOD, and CAT assessed in the gums (*p* > 0.05, Table 1). In addition, animals with PD showed greater values of TBARS (*p* < 0.05, Table 1) and a reduction in SOD values and TAC (*p* < 0.05, Table 1) in erythrocytes.

**Table 1:** Markers of oxidative damage in the gum and erythrocytes of Wistar rats with (PD) and without PD (Sham).

Tissue	Parameter	Sham (mean ± SD)	PD (mean ± SD)	*t*	*F*	*p*
Gum	TBARS (nmol MDA/mg of protein)	2.88 ± 1.01	4.44 ± 0.90	2.62	12.03	0.028*
	Carbonyl derivatives (nmol/mg of protein)	2.61 ± 1.56	2.55 ± 1.36	0.04	1.02	0.965
	Total antioxidant capacity (µmol FeSO_4_/mg of protein)	484.65 ± 194.95	1106.75 ± 218.92	3.16	6.42	0.019*
	SOD (U/mg of protein)	5.85 ± 2.76	8.67 ± 2.27	1.73	1.17	0.283
	CAT (ΔE/min/mg of protein)	3.73 ± 0.58	4.21 ± 1.36	0.40	5.74	0.705
Erythrocytes	TBARS (nmol MDA/mg of protein)	1.03 ± 0.27	1.70 ± 0.73	2.10	4.75	0.049*
	Total antioxidant capacity (µmol FeSO_4_/mg of protein)	216.28 ± 65.03	177.64 ± 48.86	2.34	4.38	0.031*
	SOD (U/mg of protein)	2.53 ± 1.27	1.18 ± 0.87	2.83	4.89	0.010*

*Significant difference assessed by an unpaired student's *t*‐test (*p* < 0.05).

CAT was lower in the hippocampus (*p* = 0.009) and higher in the amygdala of animals with PD (*p* = 0.012). No statistically significant differences were found in hippocampus and amygdala samples for SOD, TAC, TBARS, and PC (Table 2).

**Table 2:** Markers of oxidative damage in the hippocampus and amygdala of Wistar rats with (PD) and without PD (Sham).

Tissue	Parameter	Sham (mean ± SD)	PD (mean ± SD)	*t*	*F*	*p*
Hippocampus	TBARS (nmol MDA/mg of protein)	0.58 ± 0.08	0.55 ± 0.11	0.69	1.92	0.493
	Carbonyl derivatives (nmol/mg of protein)	0.88 ± 0.16	0.94 ± 0.19	0.62	1.38	0.539
	Total antioxidant capacity (µmol FeSO_4_/mg of protein)	139.05 ± 12.87	148.18 ± 11.10	1.79	2.33	0.165
	SOD (U/mg of protein)	0.16 ± 0.06	0.19 ± 0.05	1.20	1.34	0.241
	CAT (ΔE/min/mg of protein)	0.08 ± 0.01	0.05 ± 0.02	2.84	9.37	0.009*
Amygdala	TBARS (nmol MDA/mg of protein)	1.09 ± 0.12	1.07 ± 0.17	0.20	1.83	0.843
	Carbonyl derivatives (nmol/mg of protein)	0.96 ± 0.14	0.97 ± 0.20	0.09	1.63	0.921
	Total antioxidant capacity (µmol FeSO_4_/mg of protein)	131.45 ± 17.22	136.26 ± 20.71	0.46	1.44	0.644
	SOD (U/mg of protein)	0.25 ± 0.07	0.28 ± 0.07	0.81	1.06	0.423
	CAT (ΔE/min/mg of protein)	0.04 ± 0.02	0.08 ± 0.03	2.73	5.84	0.012*

*Significant difference assessed by an unpaired student's *t*‐test (*p* < 0.05).

**Figure 4:**
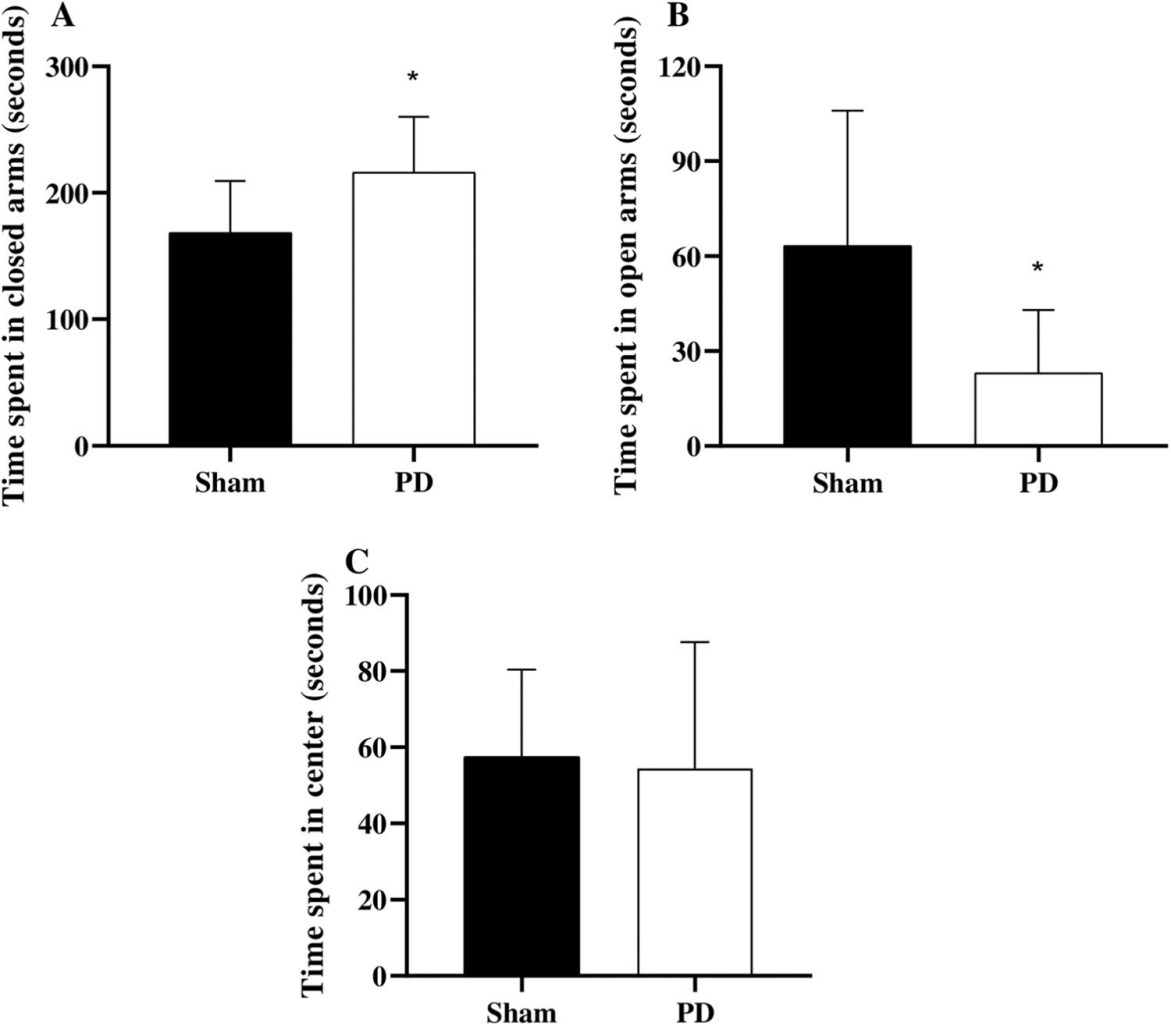
Time in the elevated plus maze test in Wistar rats with periodontal disease (PD) and without PD (Sham). (A) Time spent in the closed arms, (B) time spent in the open arms, and (C) time spent in the center. *Significant difference assessed by an paired student's *t*‐test (*p* < 0.05).

Rats with PD spent shorter time in the open arms (*p* < 0.005) and longer time in the closed arms (*p* < 0.001) of the maze compared with the sham group (Figure 4A,B). No significant differences were observed between groups in relation to the time spent in the center of the maze (*p* = 0.813). This test is used to assess anxiety‐like behavior in rodents, based on their natural aversion to open spaces. It also allows for the evaluation of spontaneous activity, which may include approach and avoidance behaviors. A longer duration spent in the closed arms and a shorter time in the open arms are indicative of anxious behavior.

**Figure 5:**
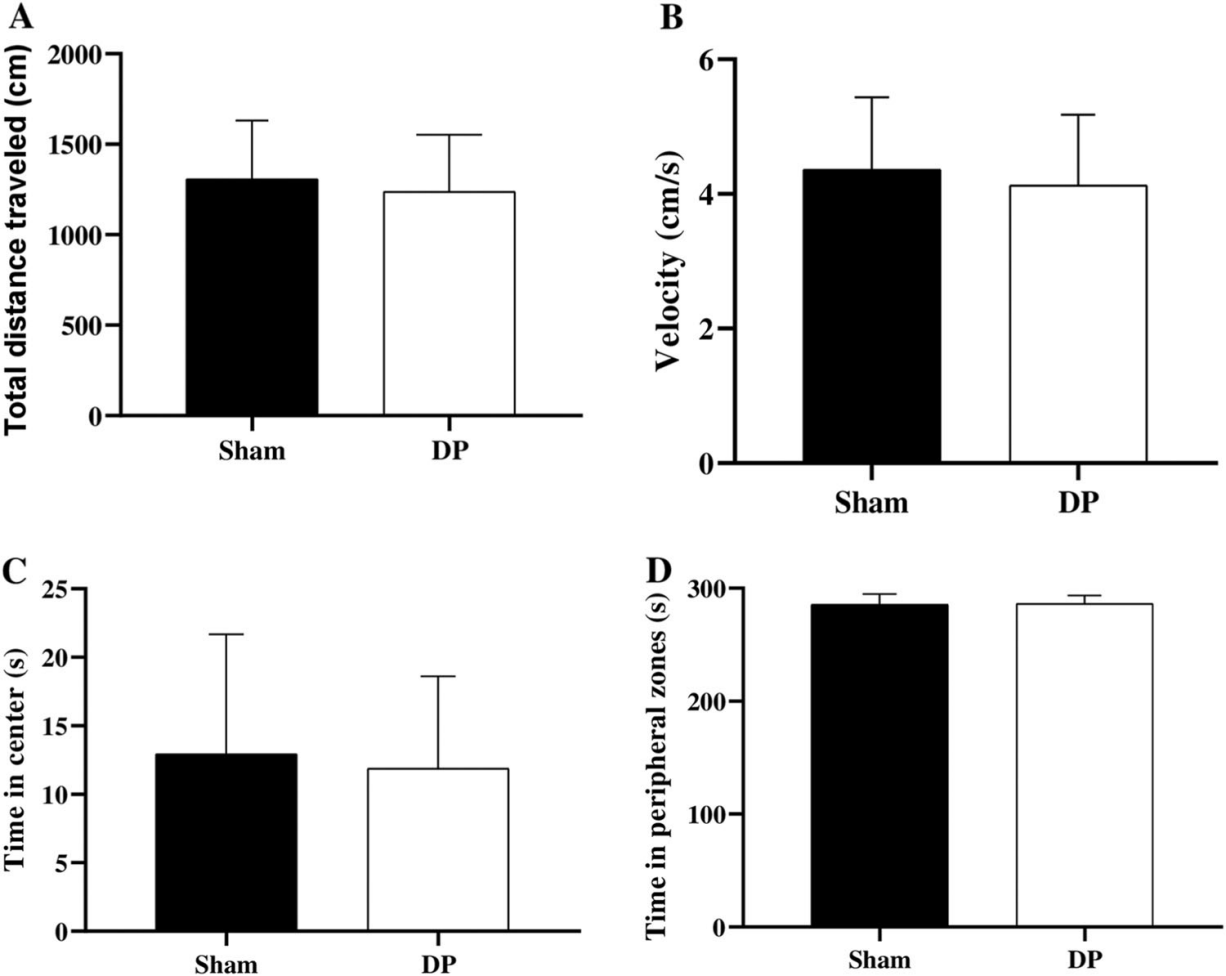
Open field test in Wistar rats with periodontal disease (PD) and without PD (Sham). (A) Total distance traveled, (B) velocity, (C) time in the center, (D) time on the peripheral zone. *Significant difference assessed by a paired student's *t*‐test (*p* < 0.05).

In the open field test, no statistically significant differences were observed in velocity (*p* = 0.529), time in the periphery (*p* = 0.864), time in the center (*p* = 0.766), and total distance covered (*p* = 0.529; Figure 5).

**Figure 6:**
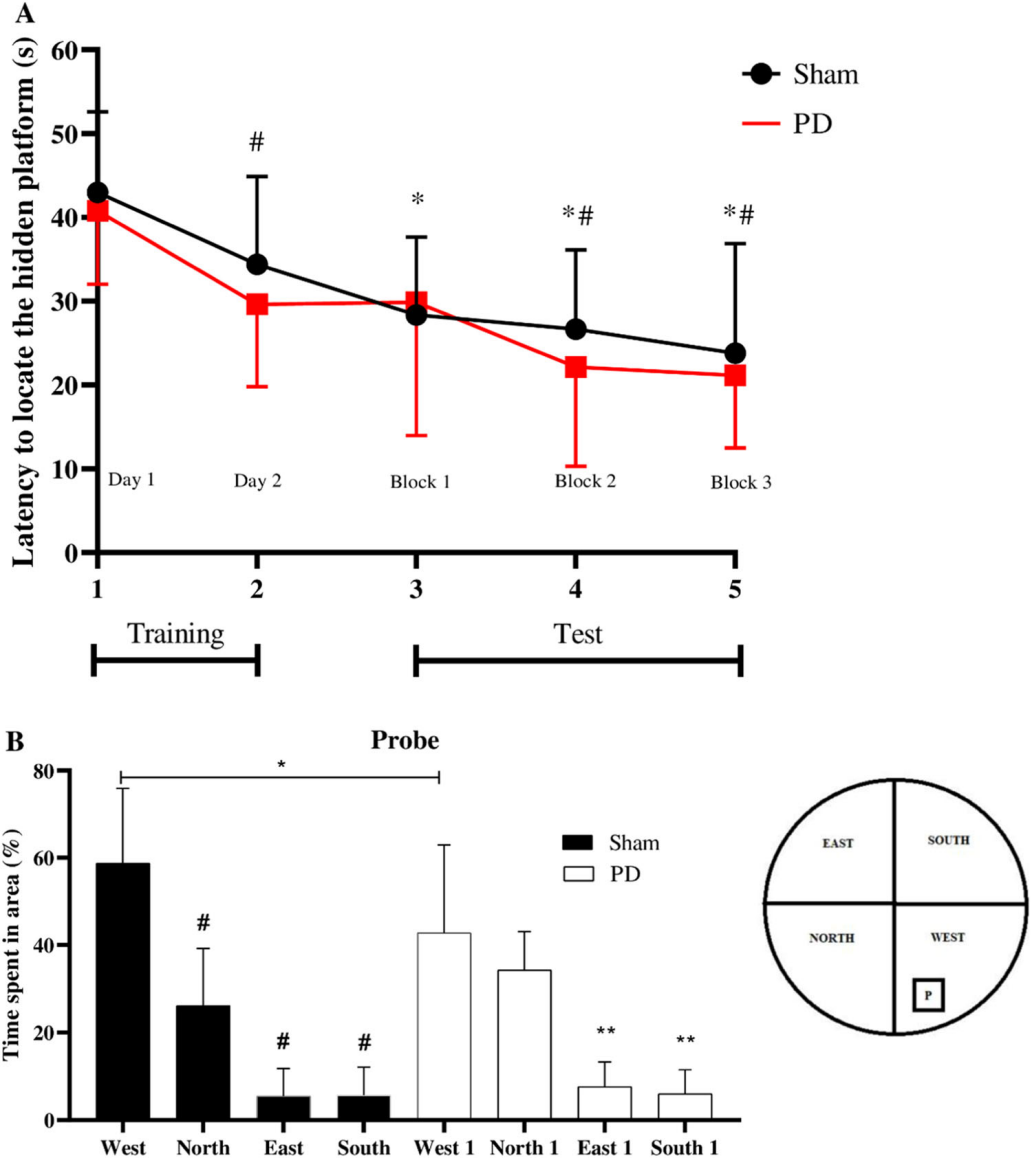
Morris water maze outcomes. (A) Days 1 and 2 (training), Day 2 (blocks 1, 2, and 3) test. Learning and spatial memory in the task of finding the platform were evaluated in Wistar rats with (PD) and without periodontal disease (sham). Statistical analysis using two‐way ANOVA with the Tukey post hoc test; *Indicates significant difference between Day 1 and block 3 in both groups (*p* < 0.05). (B) Probe test. Learning and spatial memory were assessed in the task of finding the hidden platform. Time spent in the quadrants (north, south, east, west), with west being the target quadrant. Statistical analysis using One‐way ANOVA with the Tukey post hoc test; **Indicates significant difference between groups in the west quadrant (*p* < 0.05). ^#^Indicates significant intragroup difference in relation to the west quadrant (*p* < 0.05).

In the Morris water maze, both groups showed a reduction in the latency period (between block 3 and Day 1) to find the platform (*p* < 0.05, Figure 6A). This behavior indicates an improvement in the ability to locate the platform. However, in the probe test, the animals with PD spent less time in the target quadrant (West) (*p* > 0.05), whereas those of the sham group spent more time in this quadrant (*p* < 0.001, Figure 6B). This result suggests cognitive decline, reflecting compromised learning and memory abilities in the PD group.

## 4 Discussion

Our main findings revealed an increase in anxiety‐like behavior and cognitive decline in the PD‐induced animals. Additionally, we observed significant alterations in oxidative stress parameters, both in gingival tissue and systemically, as well as in brain regions related to anxiety and cognition. Systemic redox imbalance caused by PD can also predispose to neuroinflammation and increase oxidative stress in the CNS (Chang et al. 2020). Although the CNS is protected by the blood–brain barrier (BBB), prolonged exposure to pro‐inflammatory cytokines and ROS can compromise the integrity of the BBB, allowing their infiltration into the brain (L. Liu et al. 2022). In our study, we observed that CAT levels were lower in the hippocampus and higher in the amygdala of animals with PD. Periodontal pathogens can access the systemic circulation and alter systemic inflammatory and oxidative stress mediators, affecting other tissues (Hajishengallis and Chavakis 2021; Kose et al. 2021). Additionally, these mediators can affect brain regions and disrupt the CNS homeostasis (Hajishengallis and Chavakis 2021; Kose et al. 2021). Both the hippocampus and the amygdala are affected by oxidative stress, although they may react differently due to their biochemical characteristics and neurotransmitter systems, with the hippocampus considered more sensitive to cellular damage than the amygdala (Kose et al. 2021; Cano‐Europa et al. 2008). This relationship justifies the divergent behavior of CAT in these areas found in our study. The amygdala and hippocampus are fundamental neural systems for cognition and behavior (Felger 2018), with the amygdala involved in processing emotions, whereas the hippocampus is associated with memory (Yang and Wang 2017). The contrasting alterations in oxidative stress markers evaluated in the hippocampus and amygdala of animals with PD may be associated with specific responses of each brain area to the neuroinflammation induced by PD (Wadhawan et al. 2020). Neuroinflammation is closely associated with oxidative stress, and this resultant imbalance can affect antioxidant defenses differently in the affected brain areas (Solleiro‐Villavicencio and Rivas‐Arancibia 2018). Additionally, periodontal bacteria, pro‐inflammatory cytokines, and lipopolysaccharides from PD that leak into the systemic circulation can activate the hypothalamus–pituitary–adrenal axis, increasing the secretion of hormones and neurotransmitters related to stress (Martínez et al. 2022). Although the mechanisms explaining this behavior are not well elucidated, it is possible that the greater sensitivity of the hippocampus to alterations in the stress markers may lead to a reduction in antioxidant defense in this brain area (da Silva Souza et al. 2020). The noted elevation in antioxidant activity within the amygdala suggests a potential neuroprotective response to mitigate oxidative stress. The amygdala assumes a pivotal role in emotion processing, emotional memory, and stress responses (Šimić et al. 2021). Given the amygdala's intense metabolic activity and functional demands, it is conceivable that this brain region develops a robust antioxidant capacity as an integral component of its defense mechanism against oxidative stress associated with its physiological challenges (Begega et al. 2023; Mejia‐Carmona et al. 2015). Thus, the brain regions may show distinct adaptive mechanisms in response to PD‐induced challenges (Begega et al. 2023). In this way, neuroinflammation and oxidative damage in these regions have been linked to anxiety (Felger 2018) and cognitive deficits (Li et al. 2022). Additionally, in experimental periodontitis, it has been proposed that the translocation of periodontal pathogens (e.g., *Porphyromonas gingivalis*) and their enzymes to the CNS is associated with neuroinflammation and amyloid‐beta deposition, highlighting the link between periodontitis and Alzheimer's disease (Kantarci et al. 2020).

The PD induction model used in our study has been extensively documented in the literature and has been proven to be effective in promoting bone resorption, as evidenced by greater ABL compared with the sham group. This result aligns with previous research using the ligature protocol (Andrade et al. 2017; de Molon et al. 2018). Ligature led to increased expression of the nuclear factor kappa receptor (RANKL) and a reduction in the expression of osteoprotegerin (OPG), resulting in an unbalanced RANKL/OPG ratio, ultimately contributing to increased alveolar bone resorption (Tsukasaki 2021). Furthermore, we confirmed the increase in alveolar bone resorption by observing a reduction in the percentage of calcium and phosphorus in the mandibles of animals in which PD was induced. Similar results have been observed in previous studies utilizing SEM analysis (Orlando et al. 2024; Pereira et al. 2024). This reduction in the mineral content on the alveolar bone's surface may be attributed to a pro‐inflammatory profile, where mediators such as IL‐1, IL‐6, IL‐8, and TNF‐α modulate the function of osteoclasts and osteoblasts, leading to an imbalance in bone remodeling (N. Silva, Abusleme, et al. 2015; Tsai et al. 2014). Previous studies conducted by our group observed that animals induced with periodontitis showed an increase in IL‐1 and TNF‐α expression (Andrade et al. 2017; Orlando et al. 2024; Azzi et al. 2021). These pro‐inflammatory cytokines stimulate osteoclastogenesis through the overexpression of RANK‐L, thereby promoting an increase in bone resorption (de Oliveira et al. 2024).

These changes in the inflammatory profile can lead to the translocation of cytokines and periodontal pathogens into the systemic circulation, affecting other tissues (Hajishengallis and Chavakis 2021). Additionally, inflammation in PD is mediated by oxidative stress (Sharma et al. 2021), as the activation of polymorphonucleated neutrophils, induced by periodontal pathogens, can lead to an increase in the formation of reactive oxygen species (ROS) and reactive nitrogen species (RNS) (Nguyen, Green, and Mecsas 2017). When periodontal pathogenic bacteria in biofilm trigger host defense responses, neutrophils become the predominant inflammatory cells accumulating in periodontal tissue and the gingival sulcus (Shang et al. 2023). An increased number of neutrophils are considered the primary source of oxidative stress in periodontitis, releasing excess ROS through the NADPH oxidase pathway during the phagocytosis of periodontal pathogens (Shang et al. 2023). This redox imbalance favors the destruction of the periodontium (Sczepanik et al. 2020). Furthermore, the increase in lipid peroxidation and TAC observed in animals in which PD was induced in our study aligns with findings in previous studies (Carvalho et al. 2013; Lima et al. 2017). The higher TAC in animals with PD, though unexpected, suggests a response to chronic oxidative damage. Under normal physiological conditions, there is a balance between ROS activity and antioxidant defenses. However, with challenges or widespread tissue damage, the organism can upregulate endogenous antioxidant systems to attenuate oxidative damage (Di Meo, Napolitano, and Venditti 2019). Thus, it is possible that the increase in antioxidant defenses occurs in chronic stages of the inflammatory process to respond to extensive tissue damage (Sczepanik et al. 2020; Lima et al. 2017).

The biofilm present in PD generates ROS and reduces peripheral antioxidant capacity (Brock et al. 2004). In fact, in our study, we observed significant changes in both local and systemic oxidative stress markers in animals with PD. The ROS released to combat the bacterial threat within the biofilm can lead to damage to the gingival connective tissue and the surrounding alveolar bone (Sari et al. 2021). This oxidative stress can contribute to the pathogenesis of several inflammatory diseases associated with PD (Konuganti et al. 2012). In this way, inflammation and local oxidative damage can be the trigger for systemic alterations (Sari et al. 2021), reinforcing the results of our present study, where we observed an increase in TBARS levels along with a decrease in TAC and SOD in erythrocytes of animals with PD.

The increase in ABL and disturbances in calcium and phosphorus levels in the mandibles of animals in which PD was induced serve as confirmatory parameters, attesting to the progression of PD resulting from the heightened activity of osteoclasts due to chronic inflammation (Usui et al. 2021). The locally instigated pro‐inflammatory profile has the potential to modify systemic markers, thereby influencing brain regions responsible for behavior and cognition (Li et al. 2022; Martínez et al. 2022). In our study, we hypothesized that PD could be a common causal factor for increased oxidative stress, which may contribute to altered anxiety‐like behavior and cognitive changes. Although the underlying mechanisms of this interaction were not fully elucidated in our study, our results strongly suggest a relationship between PD, oxidative stress, anxiety, and cognitive changes. In a previous study, it was observed that animals subjected to ligation for 14 days showed corticosterone levels similar to those in the control group (without ligation), whereas parameters of bone loss and pro‐inflammatory markers were higher in animals with PD (Lu et al. 2016). The findings from prior research substantiate our hypothesis that the observed alterations in the current study arise from the redox profile induced by PD, rather than from any possible physical stress resulting from ligation. Additionally, in our study, we applied the same anesthetic protocol to animals in both groups at the time of ligature placement to minimize any bias related to the side effects of the medications used. Therefore, in our experimental design, we exerted greater control over variables that could interfere with the assessed outcomes, compared with previous studies.

To support our findings of oxidative damage in the CNS areas, we conducted behavioral tests and observed that animals with PD showed greater anxiety‐like behavior and a decline in learning and memory. In the elevated plus maze test, animals with PD spent less time in the open arms and in the center, and more time in the closed arms compared with animals in the sham group. This pattern of behavior is consistent with anxiety‐like behavior (Pellow et al. 1985). Additionally, this same pattern was demonstrated in a previous study involving animals with PD induced for 14 days (Varotto et al. 2020).

In the Morris water maze, we observed a gradual reduction in escape latency during both the learning and testing phases for all animals, which indicates an improvement in the ability to locate the platform. Notably, there was a decrease in the mean escape latency of both PD and sham rats on Days 1–3. However, during the probe test, we found that animals with PD spent significantly less time in the target quadrant (West) compared with the sham group. This result suggests cognitive decline, indicating impaired learning and memory abilities in the PD group. Similar findings were reported in a study involving animals with ligature‐induced PD associated with the injection of *P. gingivalis* lipopolysaccharide (Pg‐LPS) into the gingival tissue (Qian et al. 2021). It is important to highlight that although the periodontitis induction procedure could be stressful for the animals, in this study, we also applied the anesthetic protocol to the animals in the sham group to minimize potential biases in the behavioral tests. In both experimental groups, no signs of pain or changes in body weight or food intake were observed in the animals. Additionally, from the acclimatization phase, at the beginning of the experiment, the animals were gradually adapted to the experimental room and the research team to prevent any interference with the behavioral tests. To achieve this, we made every effort to standardize the handling of the animals throughout the entire experiment.

Few experimental studies have investigated the effects of PD on cognition and behavior (Kose et al. 2021; Carvalho et al. 2013), and existing clinical studies show a high degree of heterogeneity (Zheng et al. 2021). Our results indicate that PD was associated with worse effects on anxiety‐like behavior, as well as learning and memory. Additionally, PD was related to alterations in markers of oxidative stress peripherally and in the CNS tissues analyzed. We highlight that this study is the first to explore, under a unified experimental design, the impact of PD on oxidative stress markers in the amygdala and hippocampus, while simultaneously assessing behavioral aspects in animals. Our findings are relevant for better understanding the connections between PDs and cognitive disorders. Considering the high prevalence of these conditions in the general population, the findings of the present study can contribute to a comprehensive approach to oral health and its impact on mental and cognitive health, informing healthcare practices and potentially influencing research and public health policies. Despite all precautions, our study is not without limitations. In our analyses, we did not evaluate local pro‐inflammatory cytokines that are commonly altered in periodontitis. Additionally, ABL was assessed solely through morphometry on specimens stained with methylene blue. However, complementary techniques such as micro‐CT, histomorphometry, and immunohistochemistry could have been utilized to obtain more comprehensive results for these parameters. Therefore, future studies should explore the relationship between behavioral parameters, oxidative stress, and both local and systemic inflammatory markers in an experimental periodontitis model.

## 5 Conclusion

We conclude that PD induces anxious behavior, worsens learning and memory outcomes, and alters both local and systemic oxidative stress mediators in an animal model. Future studies should consider evaluating these parameters in experimental models using female animals and/or exploring the association of PD with other diseases, such as diabetes mellitus. Additionally, future research should focus on evaluating strategies to mitigate behavioral changes and redox status in experimental models of PD.

## Author Contributions

**Giselle B. de Castro:** methodology, formal analysis, writing–original draft, writing–review and editing, visualization, and final approval of the submitted version. **Ramona R. S. Pereira:** methodology, formal analysis, writing–original draft, writing–review and editing, visualization, and final approval of the submitted version. **Caíque O. Diniz e Magalhães:** methodology, formal analysis, writing–original draft, writing–review and editing, visualization, and final approval of the submitted version. **Karine B. Costa:** methodology, writing–original draft, writing–review and editing, visualization, and final approval of the submitted version. **Etel R. Vieira:** supervision, writing–original draft, writing–review and editing, visualization, and final approval of the submitted version. **Ricardo C. Cassilhas:** conceptualization, writing–original draft, writing–review and editing, visualization, and final approval of the submitted version. **Kinulpe H. Sampaio:** formal analysis, writing–original draft, writing–review and editing, visualization, and final approval of the submitted version. **Alan R. T. Machado:** supervision, formal analysis, writing–original draft, writing–review and editing, visualization, and final approval of the submitted version. **Jaqueline do Carmo L. Carvalho:** methodology, writing–original draft, writing–review and editing, visualization, and final approval of the submitted version. **Ramiro M. Murata:** methodology, writing–original draft, writing–review and editing, visualization, and final approval of the submitted version. **Luciano J. Pereira:** supervision, formal analysis, writing–original draft, writing–review and editing, visualization, and final approval of the submitted version. **Marco F. Dias‐Peixoto:** conceptualization, writing–original draft, writing–review and editing, visualization, and final approval of the submitted version. **Eric F. Andrade:** conceptualization, supervision, formal analysis, writing–original draft, writing–review and editing, visualization, funding acquisition, and final approval of the submitted version. **Vanessa Pardi:** methodology, writing–original draft, writing–review and editing, visualization, and final approval of the submitted version.

## Conflicts of Interest

The authors declare no conflicts of interest.

## Data Availability

The data that support the findings of this study are available from the corresponding author upon reasonable request.
